# Prevalence of subclinical mastitis, related risk factors, and antimicrobial susceptibility of bacteria isolated from the milk of dairy cows in Kosovo

**DOI:** 10.17221/102/2024-VETMED

**Published:** 2025-04-28

**Authors:** Nexhat Mazreku, Driton Sylejmani, Avni Robaj

**Affiliations:** Department of Veterinary Medicine, Faculty of Agriculture and Veterinary, University of Prishtina “Hasan Prishtina”, Prishtina, Republic of Kosovo

**Keywords:** antibiotic, disc diffusion, pathogen, sensitive

## Abstract

The purpose of this study was to determine the prevalence and risk factors associated with subclinical mastitis, as well as the antimicrobial susceptibility of bacteria isolated from the milk of dairy cows in the Republic of Kosovo. The study involved 242 lactating cows from 16 farms. Data were collected through questionnaire interviews, the California mastitis test (CMT), and microbiological analysis. For the detection and identification of bacteria, conventional methods and biochemical tests were used. The disc diffusion method was used to test the susceptibility of isolated bacteria. The prevalence of subclinical mastitis (SCM) at quarter and cow level was 22.3% and 10.5%, respectively. Factors including breed, age, parity, milk production, and lactation stage were significantly correlated with the occurrence of subclinical mastitis in cows (*P* < 0.05). Major bacterial isolates were *Staphylococcus aureus* (34%), coagulase-negative staphylococci (CNS*,* 21.3%), *Escherichia coli* (18.1%), *Streptococcus uberis* (14.9%), and *Streptococcus agalactiae* (4.3%). All bacterial isolates showed high susceptibility to gentamicin, tetracycline, amoxicillin/clavulanic acid, and sulfamethoxazole/trimethoprim and low susceptibility to penicillin and streptomycin.

Mastitis is an inflammation of the parenchyma of the mammary gland irrespective of the aetiology, but the majority of causes are infectious agents (Quinn et al. 2002). Based on the manifestation of the disease, it can be classified as clinical or subclinical (Quinn et al. 2002; Abrahmsen et al. 2014). Clinical mastitis (CM) manifests as an abrupt onset of varying degrees of erythema, oedema, hyperthermia, and discomfort in the affected mammary quarter, leading to a marked decrease in milk production, thinning and discolouration of milk, presence of flocculent material, and increased body temperature (Wilson et al. 1997). The most common type of mastitis is subclinical, which is identified by a higher quantity of somatic cells in the milk without any obvious abnormalities in the udder or milk (Akers 2002).

The predominant cases of mastitis are attributed to a limited number of prevalent bacterial pathogens. The aetiology of mastitis includes the presence of contagious pathogens (*S. aureus*, *S. agalactiae*, *Mycoplasma* spp., and *C. bovis*), as well as environmental pathogens (*Escherichia coli*, *Klebsiella* spp., coagulase-negative staphylococci CNS*,* and *S. uberis*) (Cobirka et al. 2020).

Globally, subclinical mastitis (SCM) is a significant issue that costs dairy farmers money due to decreased reproductive performance, lower milk yield and quality, and veterinary expenses (Hogeveen et al. 2011).

Many studies have found that many risk factors, including age, parity, stage of lactation, milk supply, breed, previous history of mastitis, flooring type, and teat disinfection, can affect the incidence of subclinical mastitis (Karimuribo et al. 2008).

It is well-known that bacteria show antibiotic resistance at different rates, whether they come from mastitis in general or subclinical mastitis in particular. The use of antibiotics in food animal production and their improper use in treating mastitis contribute to the rise and dissemination of antimicrobial resistance (Barlow 2011; Li et al. 2018), and dairy farms may include human pathogenic bacteria that are resistant to antibiotics. Effective prevention and control of mastitis depend on timely identification and understanding of the variety of pathogens associated with the condition.

The objective of the current study was to investigate the prevalence and risk factors of SCM and the antimicrobial susceptibility of bacteria isolated from the milk of dairy cows in Kosovo.

## MATERIAL AND METHODS

A cross-sectional study was conducted between March 2023 and February 2024 involving 242 dairy cows from 16 private dairy farms in three regions (Ferizaj, Prishtina, and Prizren) of Kosovo.

During the study, only dairy farms that delivered milk to licensed dairies in the country were included, as well as only lactating cows, excluding those with visible clinical diseases, whether infectious or metabolic, as well as those treated with antibiotics and those in the dry period. Dairy farms in the three regions were randomly selected with the assistance of local veterinarians, and they used automatic milking.

The questionnaire was used to collect data from the owners and/or carers of the animals, including age, breed, parity, lactation stage, and milk production.

Based on the interview conducted, we obtained information regarding cow factors such as age (2–4, >4–7, and >7 years old), breed (Red Holstein/Frisian Holstein/others), parity (≤3 calves, 4–5, and >5), lactation stage (1–3 months, 4–6 months, and ≥7 months), milk yield (1–10 l, 11–20 l, 21–30 l, and >30 l).

### California mastitis test (CMT)

During the regular milking periods, CMT was used to screen for SCM in milk from all teats of the lactating cows. Before testing, the udder was cleaned using water and a biocide solution, and then each one was individually wiped and dried. After discarding the first stream of milk, 2 ml of milk was taken from each quarter of the udder for testing and mixed with an equivalent amount of commercial CMT reagent (RAIDEX GmbH, Dettingen on the Erms River, Germany). Mixing was accomplished by gently moving the paddle in a horizontal plane for a few seconds. The resulting mixture’s viscosity was evaluated on a scale of 0 to 4. Scores of 0 and 1 were classified as negative and trace, respectively, while scores of 2, 3, and 4 were classified as positive. If a cow tested positive for at least one quarter with a score of 2 or above, it was considered to have subclinical mastitis.

Ten ml of milk was sampled from a positive quarter in a sterile tube and kept at 4 °C until it arrived at the laboratory. Milk samples were transported within 24 h to the microbiology laboratory and immediately processed.

### Isolation and identification of bacteria

All CMT-positive quarter samples were considered for bacteriological culture by conventional methods. Initially, each milk sample was inoculated onto 5% defibrinated sheep blood agar (Liofilchem, Roseto degli Abruzzi, Italy), Mannitol salt agar (Liofilchem, Roseto degli Abruzzi, Italy), Edward’s agar (Liofilchem, Roseto degli Abruzzi, Italy), and MacConkey agar (Himedia, Thane, India), and the plates were incubated aerobically at 37 °C for 24 hours. Bacterial colonies were differentiated into Gram-positive and Gram-negative using Gram staining. Gram-positive cocci were further tested with a catalase test to differentiate the *Staphylococcus* species from *Streptococcus* species. The mannitol-positive isolates were tested by the coagulase test using rabbit plasma to differentiate *S. aureus* from other coagulase-negative staphylococci and by using the biochemical test API Staph system. Using the Edward’s agar, isolates of *S. agalactiae* (aesculin-negative, blue colonies) were distinguished from group D streptococci (aesculin-positive, black colonies). Also, the CAMP reaction and API biochemical identification strips (Bio Mérieux, Marcy-l'Etoile, France) were used for the identification of *Streptococcus* (Api-Strep). All Gram-negative bacterial isolates were subjected to an oxidase test, lactose fermentation on MacConkey agar, citrate utilisation, an indole synthesis test, colony morphology, and the biochemical test, the Analytical profile index (BioMérieux, Marcy-l'Etoile, France) for *Enterobacteriaceae* (API 20E).

### Antibiotic susceptibility test

The disc diffusion method was used to test the susceptibility of isolated bacteria against seven antibiotic discs (Liofilchem, Roseto degli Abruzzi, Italy): penicillin G (10 U), tetracycline (30 μg), amoxicillin/clavulanic acid (30 μg), gentamicin (10 μg), trimethoprim-sulfamethoxazole (25 μg), cloxacillin (5 μg), and streptomycin (10 μg). The turbidity of each bacterial inoculum was adjusted to that of the 0.5 McFarland standard and then inoculated on Mueller-Hinton agar (HiMedia, Thane, India), and for fastidious organisms such as *Streptococci*, Mueller Hinton Agar is supplemented with 5% sterile defibrinated blood.

The antibiotic discs were placed on the medium’s surface, and the bacterial plate was incubated aerobically at 37 °C for 18 hours. The zones of inhibition were measured and reported as susceptible (S), intermediate resistant (I), or resistant (R) based on the Clinical & Laboratory Standard Institute (CLSI 2017; EUCAST 2017; CLSI 2018). The reference strains, *S. aureus* ATCC 29213 and *E. coli* ATCC 25922, were used as quality control.

### Statistical analysis

The overall prevalence of subclinical mastitis was calculated by dividing the number of cows or quarters that tested positive for mastitis by the total number of cows or quarters tested. The data were entered into a Microsoft Excel spreadsheet, and statistical analysis was conducted using the SPSS software v20 (Statistical Package for the Social Sciences; IBM Corp., Armonk, NY, USA). The Chi-square (χ^2^) test was used to investigate the relationship between subclinical mastitis and risk factors (age, parity, lactation stage, breed, and milk production). An association between mastitis infection and the specific risk factor in question was deemed statistically significant if the *P*-value was less than 0.05 (*P* < 0.05). Bacterial isolates were described by frequency and percentage, and confidence intervals (95% CI) were determined.

## RESULTS AND DISCUSSION

In the present study, the prevalence of subclinical mastitis at the cow and quarter levels was 22.3% and 10.5%, respectively (Table 1), and comparatively shows a slight decrease with previous findings in Kosovo, 25.6% and 12.2% reported by Sylejmani et al. (2016). The reason for a slight decrease in the prevalence of SCM in this study probably lies in the continuous subsidisation of cow’s milk according to quality in the country, encouraging farmers to engage and take measures both in the maintenance of cows and the farm environment to increase the quality of milk.

**Table 1:** Prevalence of subclinical mastitis (SCM) at cow and quarter levels

Type of mastitis	Cows				Quarters		
	number of examined cows	SCM No.	%		number of examined quarters	SCM No.	%
Subclinical mastitis (SCM)	242	54	22.3		968	102	10.5

In the Republic of Kosovo, the Ministry of Agriculture, Forestry, and Rural Development (MAFRD) applies direct payments (subsidies) for milk according to quality categories (extra class, class I, class II, and class III).

In contrast to our findings, Hiitio et al. (2017) reported a prevalence of subclinical mastitis in Finland in 2010 of 19%, while studies conducted by Themistokleous et al. (2019) reported a prevalence in Greece of 34.5% and Sztachanska et al. (2016) in North-East Poland of 36.7%.

Out of the related risk factors considered in the current study (Table 2), age, parity, lactation stage, milk production, and breed were found to significantly (*P* < 0.05) influence the rate of prevalence of subclinical mastitis in cows. At the cow level, the most common subclinical mastitis prevalence was observed in the Holstein Friesian breed (43.1%), followed by Red Holstein (20.9%), while other breeds had the lowest prevalence (8.8%). Similarly, Taponen et al. (2016) reported a higher frequency of mastitis in the Holstein-Friesian breed. In this regard, Fesseha et al. (2021) reported that Holstein Friesian and Jersey cows have a higher susceptibility to mastitis, possibly due to high milk production. Primiparous cows had the lowest prevalence of SCM, while multiparous cows had the highest prevalence, which is consistent with the findings reported by Michira et al. (2023).

**Table 2:** Risk factors linked to subclinical mastitis prevalence in dairy cows

Risk factor	Category	Animals examined	No of animals affected	%	χ^2^	*P*-value
Age (years)	2–4	84	8	9.5	17.433	0.000 164
	>4–7	132	34	25.75		
	>7	26	12	46.1		
						
Parity	few (≤3)	108	11	10.2	45.57	0.000 0
	moderate (4–5)	119	30	25.2		
	many >5	15	13	86.7		
						
Lactation stage (months)	1–3 (early)	99	12	12.1	15.738	0.000 382
	4–6 (mid)	78	17	21.8		
	≥7 (late)	65	25	38.5		
						
Breed	Red Holstein	86	18	20.9	25.686	0.000 0
	Friesian Holstein	65	28	43.1		
	others	91	8	8.8		
						
Milk production (litres)	1–10	24	4	16.7	15.119 2	0.001 71
	11–20	110	20	18.2		
	21–30	96	22	22.9		
	>30	12	8	66.7		

A higher frequency of SCM was shown in the older cows, with those aged 8 years having the highest prevalence (42.3%), followed by those aged >5–7 (25.75%) and >2–4 years old (9.5%). The current finding was consistent with research by Nyman et al. (2007).

Other factors, such as the stage of lactation and milk production, significantly affected the prevalence of mastitis, with a higher relative frequency in late lactation (38.5%), which was in agreement with the findings reported by Kok et al. (2021). Contrary to the findings of this study, some investigations have documented a declining trend in the prevalence of SCM in cows older than eight years, possibly because the milk production of cows in this age range gradually declines, and the burden on the udder is therefore reduced (Hiitio et al. 2017).

A total of 94 different bacterial isolates were obtained from the 54 positive milk samples collected from the SCM. The current study’s findings demonstrated that the most common bacteria were *S. aureus* (34%), followed by CNS (21.3%), *E. coli* (18.1%), *S. uberis* (14.9%), *S. agalactiae* (4.3%), *S. dysgalactiae* (3.2%)*, Micrococcus* spp*.,* and *Proteus* spp. (2.1%) (Table 3).

**Table 3:** Isolated bacteria from milk samples from cows with the California mastitis test

Bacterial pathogens	No. of isolates	Isolation rate (%)	95% confidence limits
*Staphylococcus aureus*	32	34.0	24.42 ÷ 43.58
Coagulase negative staphylococci (CNS)	20	21.3	13.02 ÷ 29.58
*Eschericia coli*	17	18.1	10.32 ÷ 25.88
*Streptococcus uberis*	14	14.9	7.70 ÷ 22.10
*Streptococcus agalactiae*	4	4.3	0.2 ÷ 8.4
*Streptococcus dysgalactiae*	3	3.2	0.36 ÷ 6.7
*Micrococcus* spp.	2	2.1	0.8 ÷ 5.0
*Proteus* spp.	2	2.1	0.8 ÷ 5.0
Total	94	100.0	–

Similar findings were also observed in a previous study of subclinical mastitis in dairy cows in Sweden by Persson et al. (2011), who reported a prevalence of *S. aureus* of 31%, CNS 27%, *S. dysgalactiae* 15%, *S. uberis* 14%, *E. coli* 4.8%, and *Streptococcus* spp. 3.1%. By contrast, Holko et al. (2019), in a study conducted in Slovakia, found CNS (35.9%) as a predominant SCM pathogen, followed by *E. coli* (14.8%), *S. aureus* (12%), *S. uberis* (10.9%), and *S. agalactiae* (5.8%). Michira et al. (2023) also reported that the predominant bacterial species found in milk samples that tested positive for SCM were CNS (40.1%), *S. aureus* (15.8%), and *Micrococcus* spp. (10.4%).

The bacterial prevalence data in our study are consistent with the reasons given by the authors of previous studies. Coagulase-negative staphylococci (CNS) are known to be common in the dairy environment, which serves as a reservoir for bacteria linked to intramammary infections (De Visscher et al. 2014), and milking hygiene practices on the farm may be connected to the prevalence of *S. aureus* (Quinn et al. 2002). Additionally, environmental bacteria, including *E. coli* and *S. uberis*, were also shown to be common pathogens in dairy cattle in our study, which may be an indicator of the state of the farm environment.

According to Table 4, the isolates of *S. aureus* were most susceptible to gentamicin, sulfamethoxazole/trimethoprim, tetracycline, amoxicillin/clavulanic acid, and cloxacillin (100, 100, 90.6, 87.5, and 75%), whereas the least susceptible were to penicillin (43.75%). In the current study, similar to *S. aureus* isolates, other bacterial isolates have shown susceptibility to almost the same antimicrobials. These findings, especially the susceptibility of *S. aureus*, CNS, and *E. coli* to gentamicin, tetracycline, cloxacillin, and amoxicillin/clavulanic acid, were almost in agreement with those of the study reported by Sylejmani et al. (2016). In the previous study in Slovakia reported by Holko et al. (2019), *S. aureus* and CNS isolates also expressed more pronounced susceptibility to amoxicillin-clavulanic acid and sulfamethoxazole-trimethoprim while showing lower resistance to streptomycin and penicillin. Penicillin resistance determined in *S. aureus* and CNS isolates in our study is higher than the results from Sweden reported by Persson et al. (2011).

**Table 4:** Antimicrobial susceptibility of bacterial species isolated from milk of dairy cows with subclinical mastitis in Kosovo

Antibiotics	Number of susceptible isolates (%)			
	*Staphylococcus aureus* (*n* = 32) No. (%)	CNS (*n* = 20) No. (%)	*Escherichia coli* (*n* = 17) No. (%)	*Streptococcus uberis* (*n* = 14) No. (%)
Gentamicin (10 μg)	32/32 (100.00)	15/20 (75.00)	17/17 (100.00)	14/14 (100.00)
Tetracycline (30 μg)	29/32 (90.62)	16/20 (80.00)	13/17 (76.47)	10/14 (71.43)
Amoxicillin/clavulanic acid (30 μg)	28/32 (87.50)	16/20 (80.00)	13/17 (76.47)	10/14 (71.43)
Cloxacillin (5 μg)	24/32 (75.00)	12/20 (60.00)	7/17 (41.18)	9/14 (64.28)
Penicillin G (10 U)	14/32 (43.75)	6/20 (30.00)	2/17 (11.76)	6/14 (42.86)
Streptomycin (10 μg)	16/32 (50.00)	10/20 (50.00)	11/17 (64.70)	5/14 (35.71)
Trimethoprim/sulfamethoxazole (25 μg)	32/32 (100.00)	18/20 (90.00)	17/17 (100.00)	9/14 (64.28)

CNS = coagulase negative staphylococci

Except for streptomycin and penicillin, to which they showed minimal susceptibility, the current investigation showed that *S. uberis* expressed high susceptibility to gentamicin, amoxicillin/clavulanic acid, tetracycline, and cloxacillin (Table 4). Contrary to our findings, in a previous study in Romania, Pascu et al. (2022) reported that none of the *S. uberis* isolates showed susceptibility to tetracycline and sulfamethoxazole/trimethoprim and low sensitivity to gentamicin and amoxicillin/clavulanic acid. According to the study by Holko et al. (2019), *S. uberis* also exhibited resistance to penicillin (10.5%) and cloxacillin (42.1%), but our data indicated higher resistance to these two drugs. Our findings showed that *E. coli* isolates exhibited susceptibility to gentamicin and sulfamethoxazole/trimethoprim, followed by tetracycline and amoxicillin/clavulanic acid, and resistance to penicillin and streptomycin. In comparison with the antibiotics we tested, Holko et al. (2019) reported that *E. coli* isolates showed resistance to amoxicillin reinforced with clavulanic acid (22.1%) and streptomycin (35.1%) while showing susceptibility to sulfamethoxazole/trimethoprim. Similarly, Persson et al. (2011) also presented the susceptibility of *E. coli* to gentamicin and the resistance to streptomycin.

In conclusion, the present study showed a slight decrease in the prevalence of subclinical mastitis in dairy cows as well as an almost similar frequency of bacterial pathogens isolated from SCM from previous studies in our country, except *S. uberis,* which has shown an increase in frequency regarding the distribution of the most commonly isolated bacteria, which increases the need for better environmental conditions on dairy farms. Our findings showed that all bacterial isolates were highly susceptible to gentamicin, tetracycline, amoxicillin/clavulanic acid, and sulfamethoxazole/trimethoprim and slightly susceptible to penicillin and streptomycin.

Subclinical mastitis may be less common if good hygienic procedures are followed during the milking process and in the cow’s surroundings. CMT can be carried out frequently as a preventative step to identify subclinical mastitis early. The most effective antibiotic for a certain bacterium species can be determined by identifying the pathogenic bacterial agents and the antimicrobial sensitivity data.
